# Protograph Designing of P-LDPC Codes via M^3^ Method

**DOI:** 10.3390/e25020232

**Published:** 2023-01-27

**Authors:** Dan Song, Meiyuan Miao, Lin Wang

**Affiliations:** 1 Department of Information and Communication Engineering, Xiamen University, Xiamen 361005, China; 2 College of Telecommunications and Information Engineering, Nanjing University of Posts and Telecommunications, Nanjing 210003, China

**Keywords:** channel coding, P-LDPC code, coding optimal algorithm, graph models, bit-error ratio

## Abstract

Recently, a mesh model-based merging (M${}^{3}$) method and four basic graph models were proposed to construct the double protograph low-density parity-check (P-LDPC) code pair of the joint source channel coding (JSCC). Designing the protograph (mother code) of the P-LDPC code with both a good waterfall region and lower error floor is a challenge, and few works have existed until now. In this paper, the single P-LDPC code is improved to further verify the availability of the M${}^{3}$ method, and its structure is different from the channel code in the JSCC. This construction technique yields a family of new channel codes with lower power consumption and higher reliability. The structured design and better performance demonstrate that the proposed code is hardware-friendly.

## 1. Introduction

Channel coding is an important issue in the physical layer, which protects the source with high reliability in channel transmission. By using channel coding, the transmission rate can theoretically approach the capacity [1]. In the physical standard of fifth-generation communication, channel coding is practically implemented by the low-density parity-check (LDPC) code, which has good error-correction and anti-interference properties [2]. In this case, the LDPC code will possibly be a good candidate for sixth-generation communication.

In addition, the LDPC code is employed as the channel encoder over different transmission noises, and presents good coding performance [3]. Furthermore, the LDPC code is considered in practical environments, such as wireless body area [4,5,6] and underwater channels [7,8,9]. It is also demonstrated that the optimization of the LDPC code can further improve the coding performance.

As a capacity-approaching channel code, the protograph LDPC (P-LDPC) code provides high reliability and low power consumption in the transmission link [10]. The optimal design of the P-LDPC code can further improve the system performance [11]. In this case, different structures based on the protograph of the P-LDPC code are derived, including the protograph-based quasi-cyclic [12], the protograph-based spatially coupled [13], and the protograph-based raptor-like (PBRL) [14]. All the aforementioned structures are demonstrated with good coding performance results. In the existing fifth-generation communication, the codes in [10] are directly fabricated as the silicon chips of the channel encoder since [10] provides good “mother codes” for LDPC code constructions and expansions.

It should be noted that the “mother codes” in [10] are also called the benchmark protographs. First, the benchmark protograph provides a good protomatrix, and it can be expanded to obtain the corresponding check matrix by using the progressive edge-growth (PEG) algorithm [15]. Then, the check matrix is further designed with structural characteristics to improve the coding property. Hence, the coding property of the benchmark protograph is an important factor impacting the system performance.

For the benchmark protograph, the mainstream of coding optimization is a more structured design. This not only improves the coding property, but also it reduces the designing complexity. For example, Ref. [16] proposes the Hadamard-based P-LDPC code, Ref. [17] builds non-binary LDPC code by the graphical representation of absorbing sets, and Ref. [18] considers algebraical and graphical methods to construct rate-compatible LDPC codes. The aforementioned research works aim to improve the system performance of high reliability and low-power consumption by optimally designing the structured protographs.

However, the existing works focus on optimizing the matrices of larger dimensions. It is investigated that the majority of existing codes can outperform the benchmark code in [10] by designing high-dimension matrices, while they do not directly refresh the benchmark codes with the same dimensions. Furthermore, the high-dimension matrices will increase the code-designing complexity and storage capacity. Considering these facts, we intend to directly improve the benchmark codes with the same dimensions.

Recently, based on the graph theory, a new structure was proposed in [19], which designs a mesh model-based merging (M${}^{3}$) method to construct the double P-LDPC (DP-LDPC) code pair. Inspired by building blocks, four basic graph models are devised to merge the source and channel protographs simultaneously in [19]. In this case, the structural characteristics of both the source and channel codes are considered. Furthermore, it is demonstrated that the optimization of the DP-LDPC code pair can ameliorate the transmission performance [5,6].

To be sure, the DP-LDPC code pair is different from the single P-LDPC code. Furthermore, it is a challenge to optimally design the short-length LDPC code with both a good waterfall region and lower error floor. In addition, there are fewer works focusing on designing the mother code since its optimization is a hard task. Hence, the M${}^{3}$ method is introduced to improve the channel P-LDPC code in this paper. In doing so, the mother code in [10] can be refreshed. We aim to obtain a better benchmark protograph with both lower power consumption and higher reliability, and provide a superior basis for expanding the check matrix.

Although this work focuses on the basically theoretical research, we think that the results can be promoted to larger scopes. For functional use, the M${}^{3}$ method can be employed to optimally search the source code for realizing the source compression. For system use, the M${}^{3}$ method can be utilized both in the single channel coding and the joint source-channel coding [19]. For practical use, the M${}^{3}$ method can design the channel coding based on the LDPC code to adapt different transmission environments [4,5,6,7,8,9]. Furthermore, this work is a kind of further design based on [20], and it focuses on the detailed structure under the precondition of the matrix rank. In this case, the proposed protomatrices can be directly employed in practical application.

The key point is that the proposed channel codes refresh the “mother codes” in [10]. It should be noted that the “mother code” is the basis of different LDPC code, and it is still employed in the existing fifth-generation communication. In detail, there are three aspects of the theory. First, the M${}^{3}$ method provides an efficient way to optimally design the “mother code” with both a good waterfall region and lower error floor, and generally this is a hard task. Second, the proposed code will be a new “seed” to design various LDPC codes, including the protograph-based quasi-cyclic code, the protograph-based spatially coupled code, and the protograph-based raptor-like code. Third, the M${}^{3}$ method has universality and generality such that it will diversely promote expanding ways to obtain the objective LDPC code, which will be good candidate for the sixth-generation communication.

Overall, two contributions are summarized as follows:

(1) The existing protograph family is refreshed by the M${}^{3}$ method with both a good waterfall region and lower error floor. The proposed channel code has better performance, and it can be considered a new benchmark protograph.
(2) The M${}^{3}$ method provides a new route based on the graphical theory to effectively design the mother code with lower coding complexity, which is friendly for hardware implementation.

The rest of this paper is organized as follows. In Section 2, the system based on the P-LDPC code is introduced. The M${}^{3}$ method is detailed in Section 3, including the definitions of graph models, the protograph generation algorithm, and the searching complexity reduction. In Section 4, the simulation results about the bit error ratio (BER) and the frame error ratio (FER) are presented based on different P-LDPC codes. Section 5 concludes the paper.

## 2. System Based on P-LDPC Code

In binary field F(2), the channel coding employs a P-LDPC code to encode a block of uniformly distributed bits s. The protomatrix of the P-LDPC code is expressed as

(1) $$\begin{array}{c} B=\begin{array}{c} {\left(\begin{array}{cccc} b_{1,1} & b_{1,2} & \cdots & b_{1,n}\\ b_{2,1} & b_{2,2} & \cdots & b_{2,n}\\ \vdots & \vdots & \ddots & \vdots \\ b_{m,1} & b_{m,2} & \cdots & b_{m,n} \end{array}\right)}_{m\times n} \end{array}, \end{array}$$

where $b_{ı,ȷ}\in N$ is the degree indicating the number of linking edges between the *ı*th check node (CN) and the *j*th variable node (VN), the subscripts are $ı,ȷ\in N^{*}$, and the dimension m×n satisfies m<n and $m,n\in N^{*}$. Here, $N^{*}$ is the set of positive integers, and N is the set of natural numbers.

The code rate is defined as $R=\frac{n-m}{n-1}$ and 0<R<1. Here, the VN with the maximum column weight is punctured, which is calculated by $\max\sum_{ı=1}^{ı=m}b_{ı,ȷ}$. Then, the protomatrix B is directly expanded to obtain the corresponding check matrix H by the PEG algorithm [15]. The dimension of H is M×N and the lifting number is N/n. According to the relation between the check matrix H and the generator matrix G, it has

(2) $$\begin{array}{c} H^{T}\cdot G=0, \end{array}$$

where the generator matrix G can be calculated by the invertible H, and the dimension of G is (N−M)×N.

The encoding is realized as follows:

(3) $$\begin{array}{c} s\cdot G=e, \end{array}$$

where the length of s is N−M, and e of length *N* is the encoded sequence.

The encoded e is modulated by the binary phase shift keying scheme, and then a symbol sequence x is obtained, where the length of x is *N*. After that, x is transmitted through the additive white Gaussian noise channel as

(4) $$\begin{array}{c} y=x+n, \end{array}$$

where n is an additive noise following the Gaussian distribution of $n∼N(0,\sigma^{2})$, and the noise variance is $\sigma^{2}$.

The channel decoding is implemented by the belief propagation algorithm, which iteratively propagates the log-likelihood ratio (LLR) between VNs and CNs. First, LLR is updated from VNs to CNs as

(5) $$\begin{array}{c} L_{v\to c}=\sum_{c^{\prime }\in S\left(v\right)\backslash c}L_{c^{\prime }\to v}+L_{ch}, \end{array}$$

where the subscripts *v* and *c* represent VN and CN, respectively, S(v)\c (S(c)\v) denotes the set of neighboring CNs (VNs) of the *v* (*c*) expecting *c* (*v*). Here, $L_{ch}$ is the initial channel LLR satisfying

(6) $$\begin{array}{c} L_{ch}=\ln\frac{{\left(1+e^{\frac{-2y}{\sigma^{2}}}\right)}^{-1}}{{\left(1+e^{\frac{+2y}{\sigma^{2}}}\right)}^{-1}}=\frac{2y}{\sigma^{2}}, \end{array}$$

where y∈y.

From CNs to VNs, LLR is calculated as

(7) $$\begin{array}{c} L_{c\to v}=2\tanh^{-1}\left(\prod_{v^{\prime }\in S\left(c\right)\backslash v}\tanh\frac{L_{v^{\prime }\to c}}{2}\right). \end{array}$$

Then, LLR is summarized as follows:

(8) $$\begin{array}{c} L_{v}=\sum_{c^{\prime }\in S\left(v\right)}L_{c^{\prime }\to v}. \end{array}$$

Finally, the decoding $\hat{s}$ is determined by the soft decision of LLR as

(9) $$\begin{array}{c} \hat{s}=\left\{\begin{array}{cc} & 0,\;\;\mathrm{if}\;L_{v}\geq 0,\\ & 1,\;\;\mathrm{if}\;L_{v}<0, \end{array}\right. \end{array}$$

where $\hat{s}\in \hat{s}$, and $\hat{s}$ is the reconstructed source sequence of length N−M.

## 3. M${}^{3}$ Method

Referring to the encoding and decoding procedures, the coding property is mainly determined by the protograph and its PEG extension. In this case, the protograph improvement will obtain better system performance. To improve the channel protograph, the related techniques of the M${}^{3}$ method are introduced as follows.

The protograph is defined as a connected graph, where VN and CN are collectively called the node *v*, and the linking edge between the two nodes is signified as *e*.

**Definition** **1.** *A graph G=(V,E) is a connected graph of dimension m×n, including $\{v_{1},v_{2},\ldots ,v_{mn}\}\in V$ nodes and $\{e_{1},e_{2},\ldots ,e_{(m-1)n+(n-1)m}\}\in E$ edges. The connected graph G is expressed as a planar graph as follows:*

(10) $$\begin{array}{c} G=\begin{array}{ccccc} v_{1} & \overset{e_{1}}{↔} & v_{2} & \cdots & v_{n}\\ \;\;\;↕e_{n} & & \;\;\;\;\;\;↕e_{n+1} & & \;\;\;\;\;\;\;\;\;↕e_{2n-1}\\ v_{n+1} & \overset{e_{2n}}{↔} & v_{n+2} & \cdots & v_{2n}\\ \vdots & & \vdots & \ddots & \vdots \\ v_{mn-n+1} & \overset{e_{\left(m-1)n+(n-1)m-(n-2\right)}}{↔} & v_{mn-n+2} & \cdots & v_{mn} \end{array}, \end{array}$$

where m×n is simplified as the subscript mn, “↔” and “↕” are the nondirectional edges, V and E represent sets of nodes and edges, respectively, and $m,n\in N^{*}$.

**Definition** **2.** *The basic graph models of M${}^{3}$ method are expressed by four different planar graphs, and their dimensions are determined, as follows:*

(11) $$\begin{array}{ccc} & & G_{1}=\begin{array}{c} v_{ı,ȷ} \end{array},\;\;\; \end{array}$$

(12) $$\begin{array}{ccc} & & G_{2}=\begin{array}{c} 1\\ \;\;\;↕e_{1}\\ 0 \end{array},\;\;\; \end{array}$$

(13) $$\begin{array}{ccc} & & G_{3}=\begin{array}{ccc} 0 & \overset{e_{1}}{↔} & 1 \end{array},\;\;\; \end{array}$$

(14) $$\begin{array}{ccc} & & G_{4}=\begin{array}{ccc} v_{ı,ȷ} & \overset{e_{1}}{↔} & v_{ı,ȷ+1}\\ \;\;\;↕e_{4} & & \;\;\;↕e_{2}\\ v_{ı+1,ȷ} & \overset{e_{3}}{↔} & v_{ı+1,ȷ+1} \end{array}. \end{array}$$

Here, the basic model $G_{1}$ has one node $v_{ı,ȷ}$, and $v_{ı,ȷ}\in N$. $G_{2}$ and $G_{3}$ are filled with two nodes “0” and “1”. $G_{4}$ is a square graph which has four nodes and four edges, where $v_{ı,ȷ}$ in $G_{4}$ can take a different value from $G_{1}$. It should be noted that $G_{4}$ is a symmetric structure satisfying $v_{ı,ȷ}=v_{ı+1,ȷ+1}$ and $v_{ı,ȷ+1}=v_{ı+1,ȷ}$.

**Definition** **3.** *If a protograph G=(V,E) includes several symmetric subgraphs $G_{4}=\left(V_{4},E_{4}\right)$ satisfying $V_{4}\subseteq V$ and $E_{4}\subseteq E$, it is said that G is structured. The degree of the symmetric subgraph follows $G_{4}={\left(v_{ı,ȷ}\right)}_{2\times 2}$ for ∀$v_{ı,ȷ}=v_{ȷ,ı}$. With the number of $G_{4}$ increased, G is more structured.*

**Lemma** **1.** *A planar graph G of any dimension can be constructed by the four basic graph models of the M${}^{3}$ method. The size of G increases with the number of basic models.*

**Proof.**  Given a connected graph G=(V,E) of dimension m×n, the four basic graph models of the M${}^{3}$ method are signified as $G_{j}=\left(V_{j},E_{j}\right)$, where ȷ={1,2,3,4}. If the graph $G_{j}$ is a connected subgraph of G, it is said that G can be constructed by several $G_{j}$.

According to the definition of a connected graph [21], an undirected graph is connected if it has a path from an arbitrary node to another node. From Definition 2, the four basic models $G_{j}$ are undirected graphs. It is also obvious that the four basic models $G_{j}$ are four connected graphs.

Referring to the definition of connected subgraph [21], the node and edge sets of the subgraph should satisfy $V_{j}\subseteq V$ and $E_{j}\subseteq E$, respectively.

In Definition 1, there are mn nodes *v* and (m−1)n+(n−1)m edges *e*, where *v* and *e* are defined as $v_{k}$ and $e_{l}$, respectively, and the subscripts satisfy k∈{1,…,mn} and e∈{1,…,(m−1)n+(n−1)m}.

For $G_{1}$, it has

(15) $$\begin{array}{c} \left\{\begin{array}{c} V_{1}\left\{v_{ı,ȷ}\right\}=V\left\{v_{k}\right\}\subseteq V,\\ E_{1}\left\{\emptyset \right\}\subseteq E, \end{array}\right.\Rightarrow G_{1}\subseteq G. \end{array}$$

Here, $V_{1}\left\{v_{ı,ȷ}\right\}$ represents the node set $V_{1}$ only containing one node $v_{ı,ȷ}$, and $E_{1}\left\{\emptyset \right\}$ indicates that the edge set $E_{1}$ is an empty set.

$G_{1}$ only has one node $v_{ı,ȷ}$ which can be signified as an arbitrary node $v_{k}$ in G. No edge in $G_{1}$ is denoted as the empty set *∅*, and *∅* is a subset of G. Thus it attains that $G_{1}$ is a connected subgraph of G.

For $G_{2}$, it has

(16) $$\begin{array}{c} \left\{\begin{array}{c} V_{2}\left\{1,0\right\}=V\left\{v_{k},v_{2k}\right\}\subseteq V,\\ E_{2}\left\{e_{1}\right\}=E\left\{e_{l}\right\}\subseteq E, \end{array}\right.\Rightarrow G_{2}\subseteq G. \end{array}$$

Here, $V_{2}\left\{1,0\right\}$ represents the node set $V_{2}$ containing two nodes 0 and 1, and $E_{2}\left\{e_{1}\right\}$ indicates that the edge set $E_{2}$ only has one edge $e_{1}$.

$G_{2}$ is a column vector including two nodes and one edge. The edge $e_{1}$ in $G_{2}$ can be signified as an arbitrary $e_{l}$ in G. The two nodes span two rows; therefore, the corresponding labels are $v_{k}$ and $v_{2k}$, respectively. Thus, it attains that $G_{2}$ is a connected subgraph of G.

For $G_{3}$, it has

(17) $$\begin{array}{c} \left\{\begin{array}{c} V_{3}\left\{0,1\right\}=V\left\{v_{k},v_{k+1}\right\}\subseteq V,\\ E_{3}\left\{e_{1}\right\}=E\left\{e_{l}\right\}\subseteq E, \end{array}\right.\Rightarrow G_{3}\subseteq G. \end{array}$$

$G_{3}$ is a row vector including two nodes and one edge. Different from $G_{2}$, the two nodes span two columns; therefore, the corresponding labels are $v_{k}$ and $v_{k+1}$. Thus it attains that $G_{3}$ is a connected subgraph of G.

For $G_{4}$, it has

(18) $$\begin{array}{c} \left\{\begin{array}{c} V_{4}\left\{v_{ı,ȷ},v_{ı,ȷ+1},v_{ı+1,ȷ+1},v_{ı+1,ȷ}\right\}=V\left\{v_{k},v_{k+1},v_{2k+1},v_{2(k+1)}\right\}\subseteq V,\\ E_{4}\left\{e_{1},e_{2},e_{3},e_{4}\right\}=E\left\{e_{l},e_{l+1},e_{2l+1},e_{2(l+1)}\right\}\subseteq E, \end{array}\right.\\ \Rightarrow G_{4}\subseteq G. \end{array}$$

$G_{4}$ is a square matrix including four nodes and four edges. The nodes and edges span two rows and two columns simultaneously; therefore, the labels are signified as $v_{k}$, $v_{k+1}$, $v_{2k+1}$, $v_{2(k+1)}$ and $e_{l},e_{l+1},e_{2l+1},e_{2(l+1)}$, respectively. Thus it attains that $G_{4}$ is a connected subgraph of G.

In conclusion, the four basic models $G_{j}$ are four connected subgraphs of G. Given an arbitrary dimension, G can be constructed by using several $G_{j}$. With the number of $G_{j}$ increased, the dimension of G is enlarged. □

### 3.1. Protograph Generation Algorithm

Based on the four basic graph models, the protograph generation algorithm is proposed to construct the channel protograph, as shown in Algorithm 1.

First, an initial protograph $G_{ini}$ is given with the dimension of m×n. Each node is located by a coordinate $\left(C_{ı},C_{ȷ}\right)$, which represents the $C_{ı}$th row and the $C_{ȷ}$th column. In line 1 of Algorithm 1, the generation rules are calculated based on given m×n. Equation (25) shows the constraint of the number of using basic models $G_{j}$, expressed as $N(G_{j})$, where “⌈·⌉” and “⌊·⌋” are rounded up and down to integers, respectively. Theoretically, according to the given m×n, the maximum number of corresponding $G_{j}$ is determined as $\max N(G_{j})$.

In line 3 of Algorithm 1, three basic graph models, including $G_{2}$, $G_{3}$ and $G_{4}$, are filled in $G_{ini}$ as the coordinates change. Since the three models have different row and column dimensions, they can be distinguished during the node traversal. After padding the basic graph models, the practical number of using $G_{j}$ is counted as $N^{\prime }\left(G_{j}\right)$.

In line 4 of Algorithm 1, the satisfiability of the constructed $G_{ini}$ is determined by comparing the theoretical $\max N(G_{j})$ and the practical $N^{\prime }\left(G_{j}\right)$, following $N^{\prime }\left(G_{j}\right)\leq \max N\left(G_{j}\right)$. This ensures the objective protograph satisfying the generation rules.

In line 5 of Algorithm 1, the remaining vacancies are filled with $G_{1}$. Equation (26) calculates the number of remaining nodes. Finally, the complete protograph $G_{ini}$ is output as the objective G.

For example, the objective protograph G of dimension 3×5 is expressed as follows:

(19) $$\begin{array}{c} G=\begin{array}{ccccccccc} 1 & \; & v_{ı,ȷ} & \; & v_{ı,ȷ} & \; & v_{ı,ȷ} & \overset{e_{1}}{↔} & v_{ı,ȷ+1}\\ \;\;\;↕e_{1} & & \;\;\;\;\; & & \;\;\;\;\; & & \;\;↕e_{4} & & \;\;↕e_{2}\\ 0 & \; & v_{ı,ȷ} & \overset{e_{1}}{↔} & v_{ı,ȷ+1} & \; & v_{ı+1,ȷ} & \overset{e_{3}}{↔} & v_{ı+1,ȷ+1}\\ \;\;\; & & \;\;↕e_{4} & & \;\;↕e_{2} & & \;\;\;\; & & \;\;\;\;\\ v_{ı,ȷ} & \; & v_{ı+1,ȷ} & \overset{e_{3}}{↔} & v_{ı+1,ȷ+1} & \; & v_{ı,ȷ} & \;\;\; & v_{ı,ȷ} \end{array}, \end{array}$$

where G is combined by five $G_{1}$, one $G_{2}$, and two $G_{4}$, and $v_{ı,ȷ}\in N$ can take distinct values.

**Figure 1:**
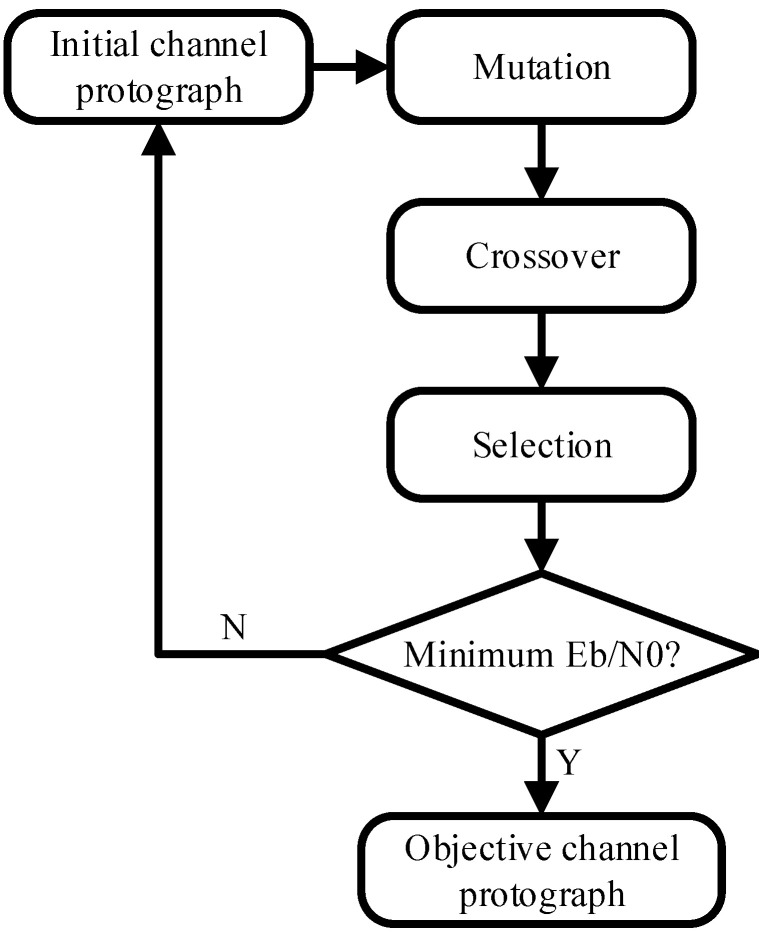
The framework of the DE algorithm.

By using the differential evolution (DE) algorithm [22], the undetermined nodes *v* are searched to match with the appropriate values. As shown in Figure 1, the initial channel protograph is iteratively updated by mutation, crossover, and selection. After an ergodic process, the objective channel protograph is determined by the objective function. The objective function is defined as

(20) $$\begin{array}{c} F=\min E_{b}/N_{0}, \end{array}$$

where $E_{b}/N_{0}$ represents the signal-to-noise ratio (SNR) in dB, and the optimization objective of function F is to achieve the minimum SNR.

Then, the protomatrix of dimension 3×5 is obtained as

(21) $$\begin{array}{c} B_{3\times 5}^{M^{3}}=\left(\begin{array}{c} \begin{array}{ccccc} 1 & 0 & 0 & 1 & 2\\ 0 & 0 & 2 & 2 & 1\\ 0 & 2 & 0 & 1 & 2 \end{array} \end{array}\right). \end{array}$$

To further expand the code rates, three larger protomatrices are acquired at different dimensions, as follows:

(22) $$\begin{array}{ccc} & & B_{3\times 7}^{M^{3}}=\left(\begin{array}{c} \begin{array}{ccccccc} 1 & 0 & 0 & 1 & 2 & 0 & 0\\ 0 & 1 & 1 & 2 & 1 & 2 & 1\\ 0 & 1 & 1 & 1 & 3 & 1 & 2 \end{array} \end{array}\right), \end{array}$$

(23) $$\begin{array}{ccc} & & B_{3\times 9}^{M^{3}}=\left(\begin{array}{c} \begin{array}{ccccccccc} 0 & 0 & 0 & 0 & 0 & 0 & 2 & 2 & 1\\ 1 & 1 & 1 & 2 & 1 & 2 & 2 & 2 & 0\\ 1 & 1 & 2 & 1 & 2 & 1 & 0 & 1 & 0 \end{array} \end{array}\right), \end{array}$$

(24) $$\begin{array}{ccc} & & B_{3\times 11}^{M^{3}}=\left(\begin{array}{c} \begin{array}{ccccccccccc} 0 & 0 & 0 & 0 & 0 & 0 & 0 & 0 & 2 & 2 & 1\\ 1 & 2 & 1 & 2 & 1 & 2 & 1 & 1 & 2 & 2 & 0\\ 2 & 1 & 2 & 1 & 2 & 1 & 1 & 1 & 0 & 1 & 0 \end{array} \end{array}\right). \end{array}$$

**Algorithm 1** Protograph generation based on graph models.**Input:** the initial protograph, $G_{ini}$; the coordinate of node, $\left(C_{ı},C_{ȷ}\right)$; the dimension of the objective protograph, m×n;**Output:** the objective protograph, G;1:Calculating generation rules based on given m×n
(25)$$\begin{array}{c} \max N\left(G_{j}\right)⇐\left\{\begin{array}{c} 0\leq N\left(G_{2}\right)<\left\lfloor \frac{m\times n}{2}\right\rfloor -\left\lceil \frac{m\times n}{4}\right\rceil ,\\ 0\leq N\left(G_{3}\right)<\left\lfloor \frac{m\times n}{2}\right\rfloor -\left\lceil \frac{m\times n}{4}\right\rceil ,\\ 0<N\left(G_{4}\right)<\left\lfloor \frac{m\times n}{4}\right\rfloor . \end{array}\right. \end{array}$$2:**for**$C_{ı}=1$ to *m* and $C_{ȷ}=1$ to *n* **do**3:   Padding basic graph models
$$\begin{array}{c} N^{\prime }\left(G_{j}\right)⇐\left\{\begin{array}{c} \mathrm{if}\;m\geq 2,n\geq 2,\;\mathrm{then}\;\left(C_{ı},C_{ȷ}\right)=\left(C_{ı+1},C_{ȷ+1}\right),\left(C_{ı},C_{ȷ+1}\right)=\left(C_{ı+1},C_{ȷ}\right);\\ \mathrm{if}\;m\geq 2,n<2,\;\mathrm{then}\;\left(C_{ı},C_{ȷ}\right)=1,\left(C_{ı+1},C_{ȷ}\right)=0;\\ \mathrm{if}\;m<2,n\geq 2,\;\mathrm{then}\;\left(C_{ı},C_{ȷ}\right)=0,\left(C_{ı},C_{ȷ+1}\right)=1. \end{array}\right. \end{array}$$4:   Decision of satisfiability: $N^{\prime }\left(G_{j}\right)\leq \max N\left(G_{j}\right)$, j∈{2,3,4}.5:   Filling vacancies by $G_{1}$
(26)$$\begin{array}{c} N^{\prime }\left(G_{1}\right)=m\times n-2\times N^{\prime }\left(G_{2}\right)-2\times N^{\prime }\left(G_{3}\right)-4\times N^{\prime }\left(G_{4}\right), \end{array}$$6:**end for**7:Output result: $G⇐G_{ini}$.

### 3.2. Searching Complexity Reduction

The measurement metric of the coding complexity is the number of searching entries. During the searching process, each node needs to match an appropriate degree. Hence, this is an exhaustive traversal method. Assuming the dimension of the objective protograph is m×n, and the range of degree is ${\left[0,3\right]}^{*}$, where ${\left[0,3\right]}^{*}$ represents the integers from 0 to 3.

In the original DE algorithm, the total number of searching entries is calculated by

(27) $$\begin{array}{c} O_{1}=4^{m\times n}, \end{array}$$

where $O_{1}$ is exponentially increased with a larger m×n.

The M${}^{3}$ method provides a more structured design of the protograph. Since there are several symmetric subgraphs and some determined nodes, the total number of searching entries is expressed as

(28) $$\begin{array}{c} O_{2}=4^{\frac{m\times n-\Phi +\Psi }{2}}, \end{array}$$

where $\frac{m\times n-\Phi +\Psi }{2}$ indicates the number of searching nodes in G, Φ represents the determined nodes consisting of $G_{2}$ and $G_{3}$, and Ψ denotes the number of remaining nodes filled with $G_{1}$. Generally, Ψ takes a smaller value.

Overall, the searching complexity is theoretically reduced to

(29) $$\begin{array}{c} \frac{O_{1}-O_{2}}{O_{1}}\approx 50\%. \end{array}$$

## 4. Simulation Results

In this section, the BER and FER performance results are compared based on different P-LDPC codes. Two benchmark codes with the same dimension as $B_{m\times n}^{M^{3}}$ are selected, namely $B_{m\times n}^{\mathrm{AR}3A}$[23] and $B_{m\times n}^{\mathrm{AR}4\mathrm{JA}}$ [24]. Furthermore, the PBRL-LDPC codes in [14] are introduced to compare with the proposed P-LDPC codes.

**Figure 2:**
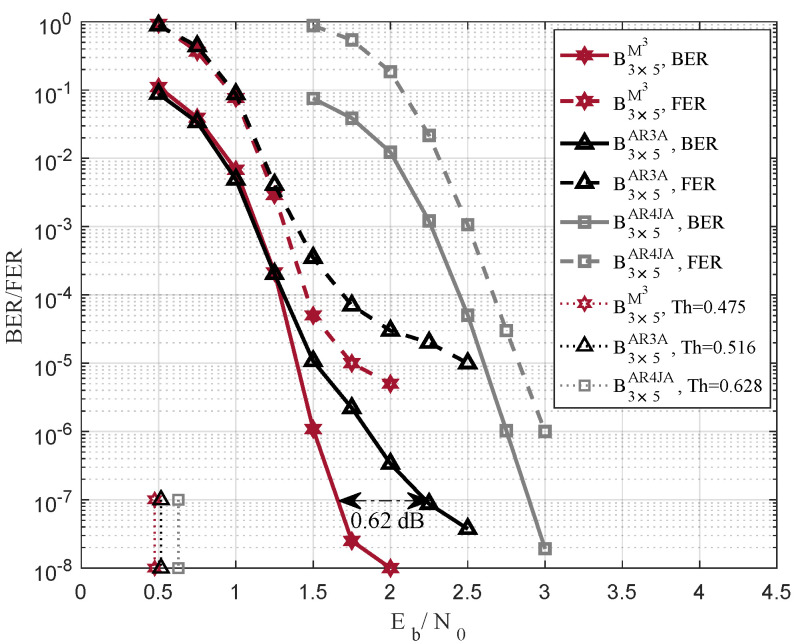
BER and FER comparisons based on benchmark codes in [10], the code rate is R=1/2, and the lifting number is 800.

Figure 2 shows the BER and FER performances compared with two benchmark codes. The code rate is R=1/2, and the lifting number is 800. The proposed $B_{3\times 5}^{M^{3}}$ in red hexagram line obtains 0.62 dB coding gains at BER = $10^{-7}$. In addition, compared to $B_{3\times 5}^{\mathrm{AR}3A}$ and $B_{3\times 5}^{\mathrm{AR}4\mathrm{JA}}$, $B_{3\times 5}^{M^{3}}$ has lower decoding threshold of Th = 0.475.

**Figure 3:**
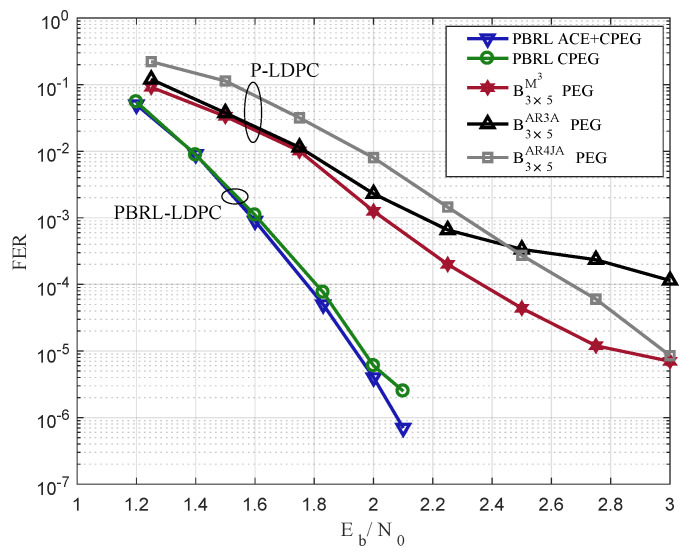
FER comparison based on PBRL-LDPC in [14] and P-LDPC codes, the code rate is R=1/2, and the lifting number is 200.

In Figure 3, the PBRL-LDPC and the P-LDPC codes are simulated by FER performance. The lifting number is 200, and the code length is 1000. For the P-LDPC type, the proposed $B_{3\times 5}^{M^{3}}$ outperforms $B_{3\times 5}^{\mathrm{AR}3A}$ and $B_{3\times 5}^{\mathrm{AR}4\mathrm{JA}}$ to present the advantage of short-to-medium length. However, two PBRL-LDPC codes [14] have better FER performance. The main reason is that the PBRL-LDPC directly optimizes the check matrix with a larger dimension, while the P-LDPC only considers the protograph of a smaller dimension.

**Figure 4:**
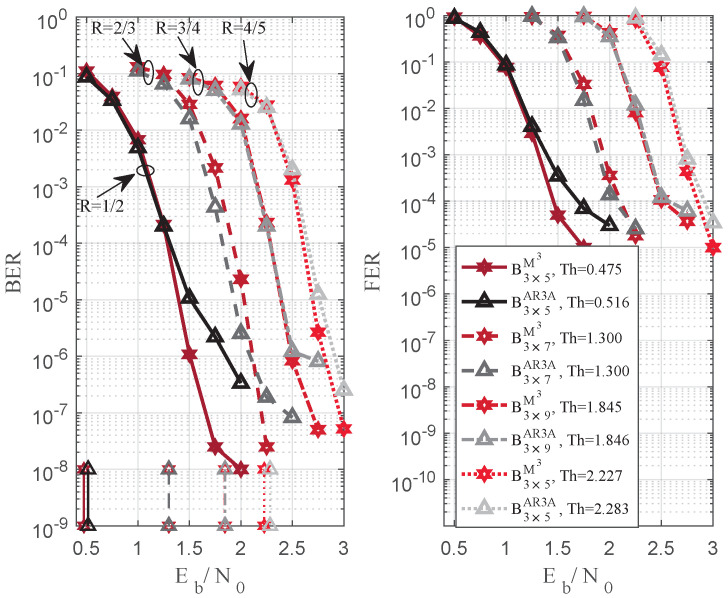
BER and FER comparisons based on $B_{m\times n}^{M^{3}}$ and $B_{m\times n}^{\mathrm{AR}3A}$ in [10], the code rate is R=1/2, 2/3, 3/4, 4/5, and the lifting number is 800.

Figure 4 demonstrates the validity of code rate extensions. The code rates are given as R=1/2, 2/3, 3/4, and 4/5, and the lifting number is 800. At the same code rate, the proposed $B_{m\times n}^{M^{3}}$ achieves a lower decoding threshold and error floor. Hence, the structured design based on the M${}^{3}$ method is effective to realize the higher reliability of the channel coding.

## 5. Conclusions

In this paper, the M${}^{3}$ method is introduced to construct the channel P-LDPC code. The structured design of the channel protograph is obtained with both a lower decoding threshold and error floor; therefore, the “mother code” in [10] can be refreshed. From this point, it is found that the optimization of the “mother code” is necessary. The proposed codes will be good candidates of the “mother code”. This structured design provides a highly symmetric protograph, which is hardware friendly in practical applications. Overall, this work cannot only be promoted to differently functional uses, including the source coding, the channel coding, the joint source channel coding, and the coding optimization over practical transmission environment, but also it has theory-driven “mother code” design. In our future work, the check matrix of the proposed protograph will be optimized by two stage extensions. The derived codes will achieve the desired performance results compared to good competitors.

## Data Availability

Not applicable.
